# The ribosome and its role in protein folding: looking through a magnifying glass

**DOI:** 10.1107/S2059798317007446

**Published:** 2017-05-31

**Authors:** Abid Javed, John Christodoulou, Lisa D. Cabrita, Elena V. Orlova

**Affiliations:** a Institute of Structural and Molecular Biology, Birkbeck College, Malet Street, London WC1E 7HX, England; b Institute of Structural and Molecular Biology, University College London (UCL), Gower Street, London WC1E 6BT, England

**Keywords:** ribosome, nascent chain, protein folding, NMR, cryo-EM

## Abstract

Protein folding, a process that underpins cellular activity, begins co-translationally on the ribosome. During translation, a newly synthesized polypeptide chain enters the ribosomal exit tunnel and actively interacts with the ribosome elements – the r-proteins and rRNA that line the tunnel – prior to emerging into the cellular milieu. While understanding of the structure and function of the ribosome has advanced significantly, little is known about the process of folding of the emerging nascent chain (NC). Advances in cryo-electron microscopy are enabling visualization of NCs within the exit tunnel, allowing early glimpses of the interplay between the NC and the ribosome. Once it has emerged from the exit tunnel into the cytosol, the NC (still attached to its parent ribosome) can acquire a range of conformations, which can be characterized by NMR spectroscopy. Using experimental restraints within molecular-dynamics simulations, the ensemble of NC structures can be described. In order to delineate the process of co-translational protein folding, a hybrid structural biology approach is foreseeable, potentially offering a complete atomic description of protein folding as it occurs on the ribosome.

## Abstract 2

The structural biology of co-translational protein folding on the ribosome is reviewed.

## 1. Introduction   

**Figure 1:**
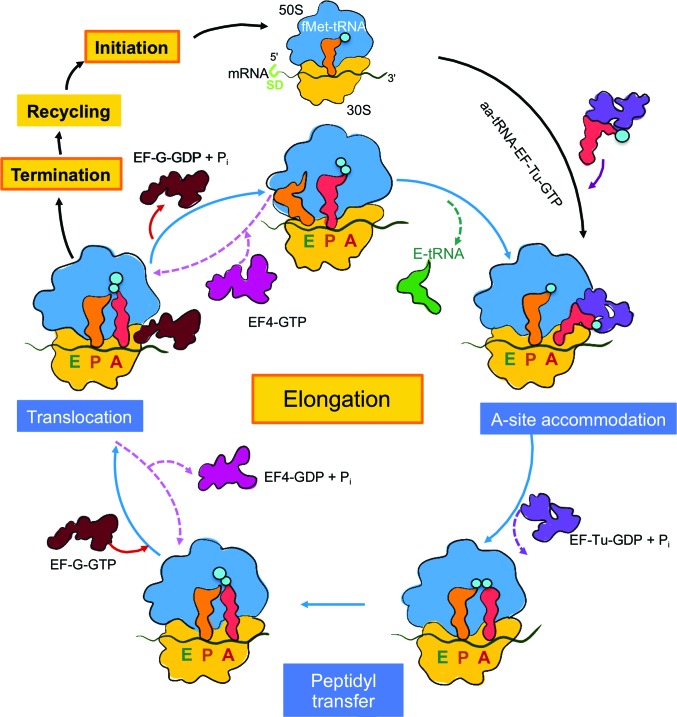
Protein biosynthesis on the ribosome. The illustrated diagram shows the key protein-translation steps performed by bacterial ribosomes. During translation, the ribosome is engaged in four key steps: initiation, elongation, termination and recycling (highlighted in yellow boxes). Translation initiation starts with the 30S subunit (yellow) binding near the initiation codon on mRNA at the Shine–Dalgarno (SD) sequence (Schmeing & Ramakrishnan, 2009 ▸; light green). Upon recruitment of the formylmethionyl-tRNA (fMet-tRNA; orange) at the P-site, carrying the methionine amino acid (cyan), the 50S subunit (blue) binds to form the initiation complex. Individual steps of the elongation cycle are shown in blue boxes. An incoming aminoacyl-tRNA (aa-tRNA; red), carrying a charged amino acid (cyan circle), bound to EF-Tu-GTP (purple) binds at the A site of the ribosome (in the A-site accommodation step). Upon mRNA decoding and a correct codon–anticodon pair between the mRNA and tRNA, EF-Tu hydrolyses GTP and dislocates (shown as a purple dashed arrow), allowing peptide-bond formation between A-site and P-site tRNAs in the peptidyl-transfer step. Elongation factor EF-G (dark brown) then binds to allow tRNAs to translocate from the A to P sites and from the P to E sites (translocation step) with energy derived from GTP catalysis. The release of EF-G (GDP-bound, shows as a brown arrow) enables deacetylated tRNA to exit (E-tRNA; green). During tRNA translocation, EF4-GTP (magenta; Qin *et al.*, 2006 ▸) can rescue stalled ribosomes by back-translocation (shown as dashed magenta arrows) to the peptidyl-transfer step to proceed with normal protein elongation.

All proteins are synthesized on the ribosome, the universal protein-biosynthesis machinery found in all kingdoms of life. The ribosome, a ribonucleoprotein macromolecular complex (ranging in size from 2.5 to 4.5 MDa), consists of two subunits that comprise ribosomal RNA (16S for small and 23S for large subunits in bacteria, and 18S for small and 28S for large subunits in eukaryotes) and ribosomal proteins (54 in bacteria and 80 in eukaryotes) (Melnikov *et al.*, 2012 ▸). This nanomachine decodes the genetic information present within a messenger RNA (mRNA) transcript and synthesizes a polypeptide chain. Protein translation by the ribosome can be divided into four main stages: initiation, elongation, termination and recycling (Fig. 1 ▸). The small subunit mediates base-pairing interactions between the mRNAs and tRNA that determine the correct amino-acid sequence of the nascent polypeptide chain, while the large subunit catalyses peptide-bond formation at the peptidyl transferase centre (PTC) between the amino acids covalently attached to tRNA during elongation (Schmeing & Ramakrishnan, 2009 ▸; Steitz, 2008 ▸; Moore, 2009 ▸). During protein biosynthesis, the nascent chain (NC) emerges vectorially (N-terminus emerging prior to the C-terminus) from the exit tunnel within the large subunit (Bernabeu & Lake, 1982 ▸; Milligan & Unwin, 1986 ▸; Yonath *et al.*, 1987 ▸), where it can begin to fold in a process described as co-translational protein folding (Netzer & Hartl, 1997 ▸).

An understanding of the molecular basis of co-translational protein folding is starting to develop: while folding is defined overall by the amino-acid sequence of a polypeptide chain, the process is further influenced by other features *in vivo* including the macromolecular crowding inside the cell, interaction with co-translational ribosome-associated factors and by the ribosome itself (Balchin *et al.*, 2016 ▸, and references therein). For these reasons, the folding pathways by which NCs acquire structure to obtain their biologically active state may be different from those observed in isolated proteins (Clark, 2004 ▸, and references therein).

Our current insight into the ribosome structure and its function has been expanded over the past two decades using high-resolution structures from X-ray crystallography and cryo-electron microscopy (cryo-EM). Moreover, the recent ‘resolution revolution’ in cryo-EM (Kühlbrandt, 2014 ▸), aided by technological developments in microscopes, data acquisition using direct electron detectors and image-processing software, has enabled the characterization of many functionally relevant ribosome complexes at near-atomic resolution (Bai *et al.*, 2013 ▸; Voorhees *et al.*, 2014 ▸; Behrmann *et al.*, 2015 ▸; Brown *et al.*, 2016 ▸). Detailed studies of the product of biosynthesis, the NC, are only beginning to emerge, following a period which was primarily focused on elucidating the structure and function of the ribosome. In recent years, a breakthrough in our comprehension of the NC has arisen *via* structural analysis of ribosome–nascent chain complexes (RNCs), *i.e.* ribosomes harbouring NCs with variable chain lengths, which enable ‘snapshots’ of biosynthesis to be taken. Advances in cryo-EM have been instrumental in showing how certain NC sequences can interact with the PTC and exit tunnel, and arrest the elongation process (Seidelt *et al.*, 2009 ▸; Bhushan *et al.*, 2011 ▸; Sohmen *et al.*, 2015 ▸; Zhang *et al.*, 2015 ▸; Arenz *et al.*, 2016 ▸). Cryo-EM of RNCs has also shown features of co-translational folding as it occurs within the exit tunnel, where NCs have been shown to form simple tertiary motifs (Nilsson *et al.*, 2015 ▸, 2017 ▸). These studies have been complemented by RNC studies using NMR spectroscopy, which is unique in its capacity to describe both the structure and the dynamic characteristics of the emerging NC as it exists beyond the tunnel and forms higher-order tertiary structure (Hsu *et al.*, 2007 ▸; Cabrita *et al.*, 2009 ▸, 2016 ▸; Cassaignau *et al.*, 2016 ▸). Together, these significant advances in methodology are bringing us closer to understanding the role of the ribosome in co-translational folding events, as they occur within cells.

This review provides a brief account of the key developments in our knowledge of co-translational protein folding and the behaviour of nascent polypeptides on the ribosome. We will highlight how structural methods of studying RNCs are being combined to provide information on the molecular mechanism by which a folding nascent polypeptide acquires structure on the ribosome inside a cell.

## 2. Structure and function of the ribosome   

### 2.1. Understanding ribosome function from structures   

A plethora of data obtained using a range of biochemical and biophysical methods have laid the foundations for subsequent structural studies. Early work using biochemical tools such as comparative DNA-sequence analysis and sedimentation equilibrium indicated that the ribosome is a complex of rRNA with higher-order structure and globular proteins (Moore *et al.*, 1968 ▸; Delius *et al.*, 1968 ▸; Noller & Herr, 1974 ▸; Herr & Noller, 1975 ▸; Brosius *et al.*, 1978 ▸). The insight into the individual components of the ribosome was a crucial benchmark for subsequent studies that detailed the overall organization and three-dimensional architecture of this fundamental nanomachine. Antibodies raised against the r-proteins enabled the localization of surface ribosomal proteins on the large and small subunits of the ribosome using immunoelectron microscopy (Tischendorf *et al.*, 1974*a* ▸,*b* ▸). Neutron scattering analysis complemented these studies of the individual subunits of bacterial ribosomes by determining the relative positions of the ribosomal proteins (r-proteins; Moore *et al.*, 1975 ▸). These observations were further supported by chemical cross-linking studies on *Escherichia coli* ribosome subunits, which provided details of r-protein–rRNA contacts (Brimacombe *et al.*, 1976 ▸). A number of early X-ray crystallographic and NMR studies made attempts to probe the structures of individual r-proteins and r-protein–rRNA interactions (Appelt *et al.*, 1981 ▸; Ramakrishnan & White, 1992 ▸; Liljas & Kurland, 1976 ▸; Kime, 1984 ▸; Zhang & Moore, 1989 ▸). Interestingly, ^1^H NMR spectroscopy (Tritton, 1980 ▸) gave an initial indication of the dynamics associated with the 70S ribosome, showing the flexibility of the stalk protein uL12 (Bocharov *et al.*, 2004 ▸; Mulder *et al.*, 2004 ▸; Christodoulou *et al.*, 2004 ▸) and the degree of disorder of the largest ribosomal protein on the small subunit, bS1 (Bushuev & Gudkov, 1988 ▸; Christodoulou *et al.*, 2004 ▸). These NMR studies revealed the dynamic regions of the ribosome that to date have been largely elusive to both X-ray and cryo-EM studies.

**Figure 2:**
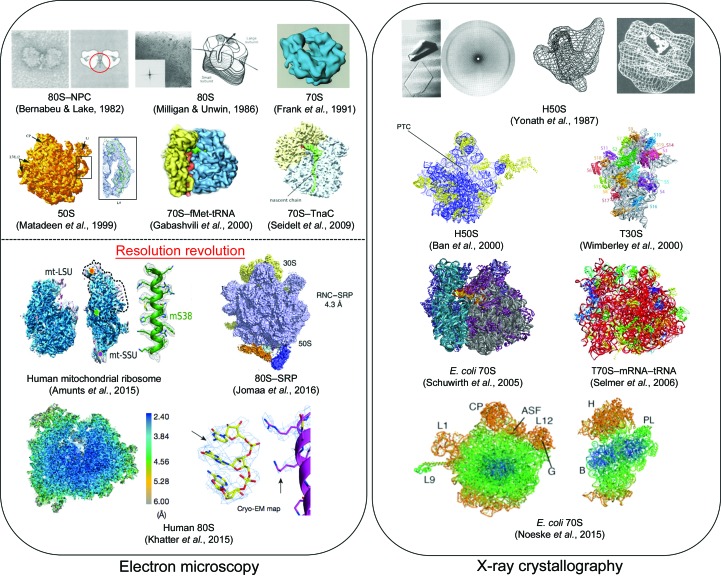
Structural biology of ribosomes. A chronological overview of structural ribosome studies related to developments in electron microscopy and X-ray crystallography. Cryo-EM has recently undergone a ‘resolution revolution’ phase (highlighted in red), revealing the structural details of ribosomes from different kingdoms of life at nearly the atomic level. The red encircled image at the upper left in the electron-microscopy panel shows the ribosome–NC complex (NC labelled with antibodies; Bernabeu & Lake, 1982 ▸), imaged using negative-stain electron microscopy to identify the relative location of the ribosome exit tunnel. In the right panel, atomic structural studies of ribosomes by X-ray crystallography are highlighted. Each structure is described in the main text.

The earlier negative-stain electron-microscopy images of ribosomes provided details of the morphology and the dimensions of both the intact particles (∼250 Å in diameter for the 70S particle and 250–300 Å for the 80S particle) and the individual subunits (Fig. 2 ▸; Lake, 1978 ▸; Bernabeu & Lake, 1982 ▸). Around this time, the existence of the exit tunnel was proposed, initially by negative-stain EM images of 80S translating ribosomes, in which β-galactosidase NCs were decorated with IgG antibodies, and subsequently by two-dimensional electron crystallography of 80S ribosomes and a low-resolution X-ray analysis of the *Bacillus stearothermo­philus* 50S subunit, which revealed a putative opening within the structures (Fig. 2 ▸; Bernabeu & Lake, 1982 ▸; Milligan & Unwin, 1986 ▸; Yonath *et al.*, 1987 ▸). This was later confirmed by cryo-EM and X-ray structures of ribosomes (Frank *et al.*, 1995 ▸; Beckmann *et al.*, 1997 ▸; Ban *et al.*, 2000 ▸; Gabashvili *et al.*, 2000 ▸, 2001 ▸).

Negative-stain EM structures of bacterial ribosomes were also able to differentiate distinct regions, in particular the central protuberance formed by the 5S RNA and r-proteins, the uL1 and uL12 stalk regions on the large subunit and the ‘head’, ‘body’ and ‘shoulder’ domains of the 16S rRNA within the small subunit. However, the limitations of negative-stain sample preparation resulted in flattened electron-density maps (Frank, 1996 ▸, and references therein).

Over subsequent years, the structures of 70S ribosome complexes obtained by EM were improved by using cryogenic methods, where embedding the particles in amorphous ice at liquid-nitrogen temperatures enabled ribosomes to be captured in a near-native environment (Dubochet *et al.*, 1988 ▸; Frank *et al.*, 1991 ▸; Matadeen *et al.*, 1999 ▸; Orlova, 2000 ▸). Indeed, one of the earliest, near-native, forms of the bacterial 70S ribosome was provided by cryo-EM (Frank *et al.*, 1991 ▸). Advances in methods for image processing (reviewed in Orlova & Saibil, 2011 ▸) enhanced the resolution of the maps. More specifically, the methods for classification of cryo-EM single-particle images highlighted an intrinsic heterogeneity within the ribosome complexes (Orlova & Saibil, 2010 ▸ and references therein). Analysis of the heterogeneity of the ribosomal complex through image classification helped to unveil key functional regions on the ribosome including the mRNA channel on the small subunit; the NC exit tunnel; binding sites for A-, P- and E-tRNAs, and their movement along the 70S ribosome during translation (Fig. 2 ▸; Frank *et al.*, 1995 ▸; Agrawal *et al.*, 1996 ▸, 2000 ▸). Consequently, it also revealed one of the characteristic ribosome motions known as the ‘ratcheting’ of the subunits as they move along the mRNA transcript during translation (Frank & Agrawal, 2000 ▸).

Simultaneously, efforts in ribosome crystallography were gaining momentum. The very first X-ray analysis of ribosome subunits derived from thermophilic and archaeal organisms, using hybrid structural tools, enabled structure determination to near-atomic resolution (Schluenzen *et al.*, 2000 ▸; Ban *et al.*, 1998 ▸). The first structure of the large subunit of *Haloarcula marismortui* ribosome (H50S) was obtained by combining the X-ray data with intermediate-resolution EM maps, which led to the subsequent high-resolution structure (Ban *et al.*, 1998 ▸, 2000 ▸). The large ribosomal subunit structure provided atomic detail of the organization of 23S and 5S ribosomal RNA with ribosomal proteins and proposed the structural basis behind the catalytic peptide-bond synthesis at the PTC (Fig. 2 ▸; Ban *et al.*, 1998 ▸, 2000 ▸). The X-ray structure of the small subunit from the eubacterial *Thermus thermophilus* 30S was pioneering in revealing the loci of the mRNA- and tRNA-binding sites, which were initially identified in low-resolution cryo-EM maps by Gabashvili *et al.* (2000 ▸), providing a structural basis for mRNA decoding (Wimberly *et al.*, 2000 ▸). At the same time, studies of the complete *T. thermophilus* 70S ribosome at high resolution allowed insight into the mRNA–tRNA binding interface between subunits, elucidating a key role for the inter-subunit RNA bridges in keeping the 50S and 30S intact during protein translation (Yusupov *et al.*, 2001 ▸).

Crystallographic analyses based on the seminal studies described above resolved the structures of intact ribosomal complexes in a range of functional states. These highlighted key aspects of the conformational changes in the small subunit responsible for mRNA decoding during complementary base pairing (Vila-Sanjurjo *et al.*, 2003 ▸), the possible helicase activity of the ribosome as it decodes mRNA (Takyar *et al.*, 2005 ▸) and the structural basis for mRNA binding to 30S during translation initiation, as well as the movement of mRNA along the ribosomal particle during translation (Yusupova *et al.*, 2006 ▸). The importance of the role of solvent molecules in interaction with ribosome substrates, retaining the structural integrity of the ribosome and its ‘ribozyme’ activity, was highlighted in the complete atomic structure of a 70S–mRNA–tRNA *T. thermophilus* ribosome complex (Selmer *et al.*, 2006 ▸). The structure provides details, for example, of the role of magnesium ions in coordination of the interaction of the ribosome with mRNA and with the A-site, P-site and E-site tRNA molecules during protein translation (Selmer *et al.*, 2006 ▸).

As the X-ray analysis of ribosomes progressed at the beginning of the new millennium, ribosome crystallography shifted from resolving archaeal and thermophilic bacterial ribosomes to mesophilic bacterial ribosomes. This is exemplified by the analysis of the *E. coli* 70S ribosome, where the X-ray structure provided a molecular basis for complex assembly and inter-subunit movement during translation; contacts were observed at the interface between the large and small subunits, which are mediated by several inter-subunit RNA bridges (Schuwirth *et al.*, 2005 ▸). In cryo-EM, motion of ribosome subunits was indicated by Valle *et al.* (2003 ▸). A comparative analysis of the two independent copies of the ribosome that were present in one asymmetric unit indicated that the ribosomes adopt different conformations reflecting movements of mRNA and tRNA on the small subunit during translocation. The structures also revealed conformational differences around the PTC area and ultimately provided an early structural insight into the different functional states possible for the ribosome during translocation (Schuwirth *et al.*, 2005 ▸). Later, the resolution was improved to 2.4 Å in the X-ray structure of the *E. coli* 70S ribosome, revealing conservation in the ribosome subunit interface and providing a structural basis for the importance of coordination of the bacterial *E. coli* ribosome elements by solvent molecules to retain ribosome structural integrity (Noeske *et al.*, 2015 ▸). The structure also indicated rRNA nucleotide modifications around the PTC, suggesting an important functional role in ribosome–A-site tRNA inter­actions, an aspect which is poorly understood and is open to future research. Cumulatively, these and many other major accomplishments in X-ray and EM analyses of the ribosome not only helped to rationalize much of the previously deduced experimental data on ribosome structure and function but have also opened many new avenues for exploration. This is elegantly demonstrated by the recent advances in time-resolved cryo-electron microscopy, based on insights from single-molecule fluorescence measurements. The studies provided a detailed insight into the structure of the ribosome in real time as it transitions through different functional states during protein biosynthesis (Fischer *et al.*, 2010 ▸; Tsai *et al.*, 2014 ▸; Chen *et al.*, 2015 ▸; Belardinelli *et al.*, 2016 ▸).

The developments in cryo-EM structure analyses and also in preparative biochemistry are now allowing researchers to obtain near-atomic structures of eukaryotic and mammalian ribosome complexes, and thus serve to further expand our understanding of ribosome function. The remarkable achievements include the structure of the 55S human mitochondrial ribosome complex, which strikingly differs in structural morphology from bacterial ribosomes and eukary­otic cytosolic ribosomes, exhibiting unique differences such as the presence of specific mitochondrial ribosome proteins (Amunts *et al.*, 2015 ▸; Noeske *et al.*, 2015 ▸; Ben-Shem *et al.*, 2010 ▸). Interestingly, a comparison of the large ribosomal subunits from human, porcine and yeast mitochondria with the bacterial 50S subunit revealed differences in the position of the exit tunnel site (Amunts *et al.*, 2015 ▸; Greber *et al.*, 2014 ▸; Amunts *et al.*, 2014 ▸). The boundaries of the tunnel, defined by loop extensions of the ribosomal proteins uL22, uL23 and uL24, form a different path within the yeast mitochondrial ribosome, located ∼35 Å away relative to the location expected in bacterial ribosomes (Amunts *et al.*, 2014 ▸). The mitochondrial ribosome exit tunnel is also wider than in bacterial ribosomes by ∼15 Å, which is likely to have implications for the co-translational folding behaviour of an emerging NC (Greber & Ban, 2016 ▸).

The ‘resolution revolution’ in cryo-EM enabled Khatter and coworkers to resolve the complex architecture of the human 80S ribosome, one of the largest ribosomes (Khatter *et al.*, 2015 ▸). This structure showed key differences from the yeast 80S ribosome and *E. coli* 70S ribosome and also uncovered potential eukaryotic specific antibiotic-binding sites (Ben-Shem *et al.*, 2010 ▸; Noeske *et al.*, 2015 ▸; Khatter *et al.*, 2015 ▸). The identification of such structural differences between ribosomes from different organisms (*e.g.* eukaryotic *versus* prokaryotic) is essential for improving the understanding of antibiotic selectivity (Wilson, 2014 ▸). More recently, subtle differences have been uncovered between human ribosomes (*e.g.* cytosolic *versus* mitochondrial ribosomes), which reveal novel ligand-binding sites that may be a means of devising novel therapies to specifically target cancerous human cells (Myasnikov *et al.*, 2016 ▸). In addition, understanding quality control at the level of the ribosome has come to the fore, as revealed by cryo-EM structures of the ribosome quality-control complex (Shao *et al.*, 2015 ▸) and no-go mRNA decay complexes (Becker *et al.*, 2011 ▸; Schmidt *et al.*, 2016 ▸). This momentum in the high-resolution structural characterization of ribosomes from different kingdoms of life is elucidating the intricacies of ribosome function.

### 2.2. The ribosome exit tunnel: a site for elongation regulation and co-translational folding   

**Figure 3:**
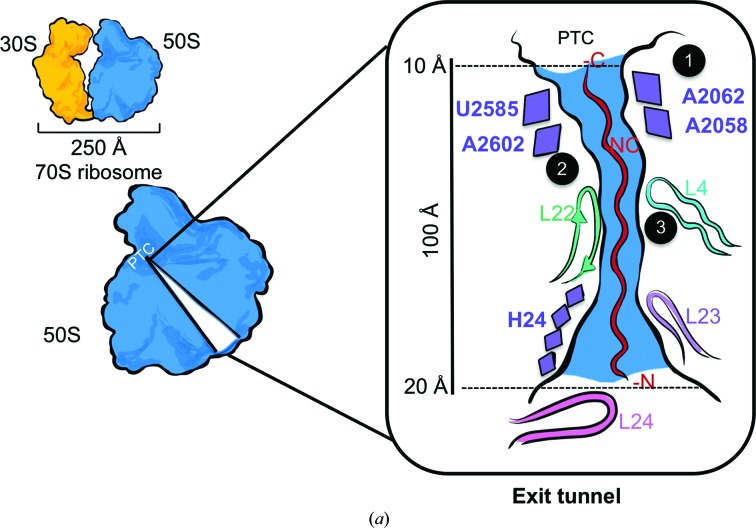

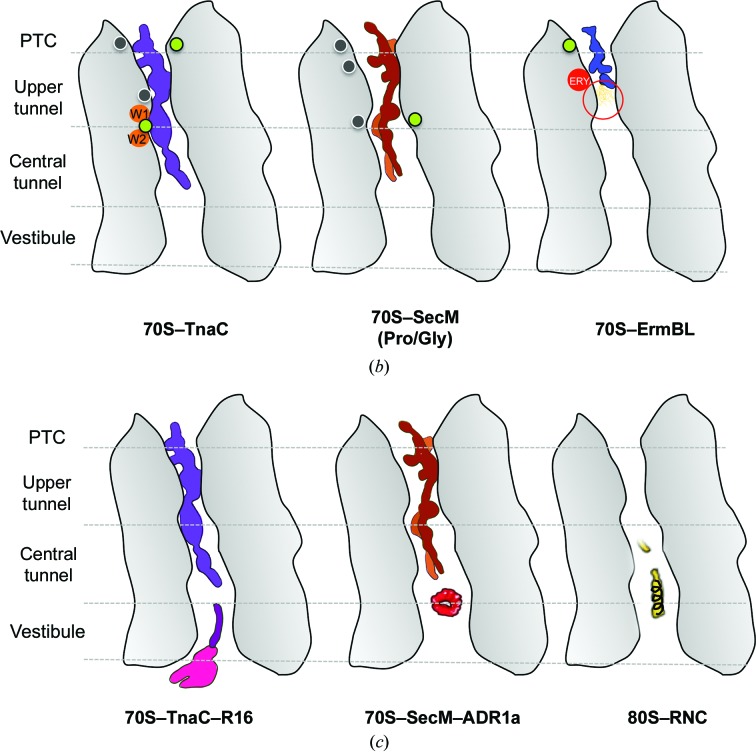
The ribosomal exit tunnel and NCs visualized by cryo-EM. (*a*) The active ribosome comprises 30S (yellow) and 50S (blue) subunits. The exit tunnel site is shown in the central section of the large 50S subunit. The tunnel starts at the PTC and is lined with 23S rRNA nucleotides (purple), the L4 and L22 loops (cyan and green), forming a constriction site, the L23 (violet) loop, 23S rRNA nucleotides (purple) and the L24 (pink) loop at the vestibule region and is shown here with a nascent polypeptide chain (red). The dimensions of the exit tunnel are narrower at the top, ∼10 Å (starting at the C-­terminus of the NC), and wider near the vestibule, ∼20 Å. 23S rRNA nucleotides and constriction-site residues (marked regions 1–3, respectively) interact equally with the NC. (*b*) A schematic representation of the three (bacterial) ribosome-stalling NCs visualized by cryo-EM. The left sides of (*b*) and (*c*) indicate different areas in the tunnel (starting at the PTC): upper, central tunnel and vestibule regions. Types of interactions between the tunnel components and the stalling NC residues and their relative interaction points are indicated in different colours (grey circle for non-electrostatic, green circle for electrostatic). l-­Tryptophan-binding pockets and an antibiotic-binding pocket for ERY are shown in orange and red, respectively. In 70S–TnaC (shown in purple), the Pro24 and Val20 residues of the TnaC NC interact with U2585 (grey circle) of 23S rRNA, Lys18 interacts with A2058 (green circle), Phe11 interacts with A751 of 23S rRNA (grey circle) and Trp12 interacts with L22 Lys90 (green circle), requiring free l-tryptophan (W1 and W2, orange) molecules to induce ribosome stalling. 70S–SecM shows two SecM NC conformations: SecM-Pro (opaque brown) and SecM-Gly (brown) stalled forms. In SecM-Gly, Ala164 interacts with U2585 (grey circle), Arg163 interacts with the U2585 nucleotide of 23S rRNA (green circle) and Trp155 interacts with Arg64 of the L4 loop (green circle) or A751 of 23S rRNA (grey circle) in SecM-Pro, to induce ribosome stalling. In 70S–ErmBL, the NC (in blue) also adopts a unique conformation induced by bound antibiotic erythromycin (ERY, red) to induce ribosome stalling. The flexible N-terminal residues (shown in yellow, encircled in red) do not interact with ERY but instead adopt altered geometry to allow the critical C-terminal Arg7 residue to interact with U2586 of 23S rRNA (green circle) and cause a translational pause. (*c*) Three NCs co-translationally folding at the vestibule region on stalled ribosomes as visualized by cryo-EM. 70S–TnaC–R16 (TnaC in purple, GS linker in dark purple, R16 in pink) shows the R16 partially folded domain at the lower vestibule region. 70S–SecM–ADR1α (SecM in brown, ADR1α in red) shows the folded zinc-binding domain at the vestibule region of the tunnel. In 80S–RNC, on a non-stop codon mRNA stalled ribosome, the NC forms an α-helix (in yellow with the α-helix shown as a black line) at the start of the vestibule region.

Protein elongation is a dynamic process and requires the ribosome to be able to interact with a range of substrates (Fig. 1 ▸); the most important amongst them are the NCs, each of which is unique in its amino-acid composition and its capacity to form structure. For this purpose, the ribosome has evolved an exit tunnel to direct the growing NC into the cellular milieu (Fig. 3 ▸ *a*). In bacteria, the ribosomal exit tunnel is ∼100 Å in length, with an average diameter of 15 Å (it varies from 10 Å at the P-site tRNA-binding site to ∼20 Å at the widest part of the exit vestibule; Fig. 3 ▸ *a*). The average diameter of the channel is said to be sufficient to accommodate water molecules and ions as well as to support some forms of NC structure, such as α-helices (Nissen *et al.*, 2000 ▸; Voss *et al.*, 2006 ▸). The tunnel, which is composed of both rRNA and r-proteins, can be divided into three regions: the upper region contains the key nucleotides U2585 and A2062 from domain V of the 23S rRNA, which interact with the NC at the tunnel entrance (Fig. 3 ▸ *a*; Voss *et al.*, 2006 ▸), the central tunnel region is constricted by the uL4 and uL22 protein loops ∼45 Å from its entrance (Fig. 3 ▸ *a*), and the lower region is formed by nucleotides from domains III and I of 23S rRNA and loops from uL23 and uL24 that line the vestibule region (Fig. 3 ▸ *a*; Nissen *et al.*, 2000 ▸).

Originally, the ribosome tunnel was considered to be a passive conduit for NCs, but more recent analyses indicate an active role in the earliest stages of protein biosynthesis, as it ‘senses’ the passage of NCs. It orchestrates co-translational events including translational arrest at the elongation step of protein biosynthesis (Nakatogawa & Ito, 2001 ▸; Murakami *et al.*, 2004 ▸) and limited folding of the NCs (Cabrita *et al.*, 2016 ▸; Nilsson *et al.*, 2017 ▸), and represents a major hub for the recruitment of molecular chaperones, NC-modifying enzymes and the translocation machinery (Kramer *et al.*, 2009 ▸; Balchin *et al.*, 2016 ▸).

## 3. Co-translational protein folding   

### 3.1. What, where, how: nascent chains folding co-translationally   

From the wealth of folding studies of isolated proteins over several decades, it has been established that the amino-acid sequence directs the folding process, which occurs along a biased energy landscape (Anfinsen, 1973 ▸; Bryngelson *et al.*, 1995 ▸). Coinciding with the structural studies of the ribosome at the time, a number of early biochemical studies used limited proteolysis to probe the structure of the growing NC on ribosomes (Blobel & Sabatini, 1970 ▸; Malkin & Rich, 1967 ▸; Protzel & Morris, 1973 ▸), yet for several decades afterwards studies of the NC remained sparse. A subsequent renewed interest resulted in a number of seminal studies of the co-translational folding of NCs within the cellular context, showing that attached NCs can acquire biological activity and be recognized by conformational antibodies and enzymes (Nicola *et al.*, 1999 ▸; Frydman *et al.*, 1999 ▸; Komar *et al.*, 1993 ▸; Tsalkova *et al.*, 1998 ▸; Clark & King, 2001 ▸; Cabrita *et al.*, 2010 ▸ and references therein). A range of structural and biophysical studies have indicated that certain NCs can form secondary-structure and even simple tertiary-structure motifs within the ribosome exit tunnel: the dimensions of the exit tunnel permit the formation of α-helices within the central and lower tunnel regions, the formation of a small zinc-finger motif at the lower tunnel region and the formation of a β-hairpin motif of transmembrane helices at the vestibule (Woolhead *et al.*, 2004 ▸; Lu & Deutsch, 2005 ▸; Kosolapov *et al.*, 2009 ▸; Bhushan *et al.*, 2010 ▸; Nilsson *et al.*, 2015 ▸). While the ∼20 Å width of the ribosome exit tunnel vestibule seems to preclude the formation of higher-order tertiary structure, simple tertiary-structure formation for smaller proteins has been found to be possible, such as a partially folded three-helix bundle at the exit of the vestibule (Nilsson *et al.*, 2017 ▸).

Structural observations such as these have also prompted investigations to dissect the earliest stages of NC folding. More specifically, biophysical experiments demonstrate that co-translational folding of HemK NCs can involve initial compaction of NCs occurring within the tunnel as a means of promoting folding (Holtkamp *et al.*, 2015 ▸). Such NC compaction may guide the consecutive folding steps, as observed in the multi-domain CFTR NCs (Kim *et al.*, 2015 ▸). In contrast, autonomous folding of individual domains has been observed in NMR studies of the NC of a multi-domain filamin protein (Cabrita *et al.*, 2016 ▸). Cumulatively, these studies are beginning to reveal the intricate molecular details and diversity associated with the folding pathways that are accessible to the emerging, ribosome-bound NCs.

### 3.2. The role of the ribosome in nascent chain folding   

Understanding the role of the translating ribosome in modulating the NC folding process is a crucial step towards revealing the early steps of protein folding as it occurs inside cells. Simulations studies suggest that nearly one-third of cytosolic proteins can exhibit co-translational protein folding (Ciryam *et al.*, 2013 ▸). The fraction of folded proteins can be related to the average rate of protein translation (2–20 amino acids per second in *E. coli*; Young & Bremer, 1976 ▸), which is typically slower than the rate of folding for small proteins (Dobson, 2003 ▸).

**Figure 4:**
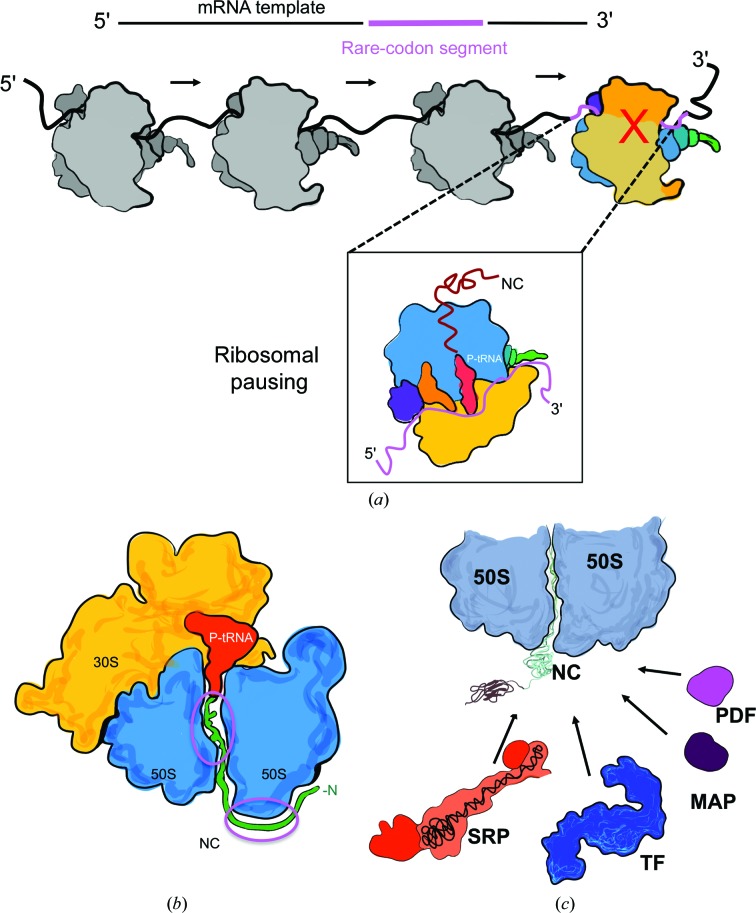
The participation of ribosome in co-translational protein folding. (*a*) An mRNA template with synonymous codons (in black) often includes substituted rare-codon clusters (in pink) near the 3′ end. Ribosomes (dark grey) progressively translate (indicated by black arrows) NCs using this mRNA template until they encounter the rare-codon segment, where they pause (ribosome in colour with a red cross). The paused ribosome state (boxed) provides time for the emerged NC (in red) to undergo folding. (*b*) An NC sequence (shown in green) can interact with the ribosome surface inside and on the outside surface (highlighted in pink circles), which can help NCs to avoid misfolding. (*c*) Several ribosome-associating factors (RAFs) bind to the ribosome co-translationally and interact with the emerging NC (only bacterial RAFs are shown). N-terminal processing RAFS (PDF, magenta; MAP, purple) bind to the ribosome near the exit port of the 50S. Similarly, chaperones such as SRP (in orange; RNA in black) and TF (in blue) bind near the 50S exit port to assist co-translational translocation and folding, respectively.

The rate of protein synthesis can be attenuated at the mRNA level by the substitution of synonymous codons for ‘rare’ codons (Fig. 4 ▸ *a*; Zhang *et al.*, 2009 ▸). Rare codons can exist in clusters that cause the ribosome to pause translation (Komar *et al.*, 1999 ▸). The clusters are also suggested to be present at protein domain boundaries, which has implications for the folding of multi-domain proteins; transient translational pausing between domains was found to be essential in the folding of the *E. coli* protein SufI (Zhang & Ignatova, 2011 ▸). This type of transient pausing is said to be favourable for ensuring that efficient co-translational protein folding takes place by allowing segments of NCs to fold before the complete emergence of the full protein (Komar *et al.*, 1999 ▸; Clarke & Clark, 2008 ▸).

During biosynthesis, the NC emerging out of the exit tunnel can interact with the ribosome surface, which is also likely to influence NC structure formation (Fig. 4 ▸ *b*). As suggested by fluorescence anisotropy experiments on RNCs of the dis­ordered protein PIR, NC interactions with the ribosomal surface can be mediated by electrostatics (Knight *et al.*, 2013 ▸). Similar interactions between the NC and the ribosome have also been observed using intrinsically disordered α-synuclein RNCs (Deckert *et al.*, 2016 ▸), suggesting that electrostatically mediated interactions are likely to be a common feature in modulating co-translational folding for an NC. More broadly, interactions with the ribosome surface that modulate the kinetic rates of folding and favour native structure formation have been demonstrated by optical tweezer experiments on T4 lysozyme NCs (Kaiser *et al.*, 2011 ▸). In addition, NMR studies of a multi-domain filamin protein show that the ribosome ‘delays’ the folding of a tandem pair of immunoglobulin domains (Cabrita *et al.*, 2009 ▸, 2016 ▸). These studies strongly indicate that the ribosome surface plays a role in preventing NC misfolding and aggregation by providing a protective local environment for correct folding to take place.

NCs emerging from the ribosomal exit tunnel encounter and interact with a range of ribosome-associated protein factors and molecular chaperones (Fig. 4 ▸ *c*). Ribosome-associated proteins that act co-translationally on the NC include peptide deformylase (PDF), methionine aminopeptidase (MAP), signal recognition particle (SRP) and trigger factor (TF). The function of these factors ranges from N-terminal NC processing to assisting co-translational folding and translocation to membrane compartments. Coordination between ribosome binding and the function of these factors plays an apparent role in co-translational protein folding (Sandikci *et al.*, 2013 ▸).

Structural analysis of these transiently binding factors has proven to be challenging. Nevertheless, the evolution of cryo-EM has opened opportunities to study the dynamic interplay between the NC and auxiliary factors such as TF, the dimeric molecular chaperone that binds as a monomer to the ribosome at the tunnel exit *via* uL23 (Kramer *et al.*, 2002 ▸). A recent high-resolution cryo-EM structure of 70S–TF–NC provided structural insights into the degree of flexibility in TF domains and the NC binding specificity when interacting with an emerging NC on the ribosome (Deeng *et al.*, 2016 ▸). The SRP is another protein which competes for the NC *via* the uL23 binding site (Schibich *et al.*, 2016 ▸) and recognizes the N-terminal signal peptide sequence on NCs to initiate protein translocation. Recent cryo-EM analysis of the 70S–SRP complex have captured intermediate states of SRP bound to the 70S ribosome, providing snapshots of SRP engagement with the ribosome-emerging NC (Jomaa *et al.*, 2016 ▸). This occurs in a co-translational manner, before targeting the NC to the membrane compartment of the cell (von Loeffelholz *et al.*, 2015 ▸; Jomaa *et al.*, 2016 ▸;). These recent accomplishments in the structural analysis of dynamic ribosome complexes demonstrate how increasingly complex questions related to ribosome function are no longer beyond the reach of structural biology.

### 3.3. Structural analysis of ribosome-bound nascent chains   

Methodological advances in both preparative biochemistry and high-resolution structural methods have enabled significant progress in illuminating the behaviour of NCs both inside and as they emerge from the ribosome exit tunnel. In particular, cryo-EM and NMR spectroscopy have facilitated direct structural, dynamic and functional characterization of the newly translated nascent polypeptides, as described below.

#### 3.3.1. Visualization of nascent chains within stalled ribosomes   

To study the interactions between the ribosome and an elongating NC, it is necessary to synchronize the actions of the ribosomal population by arresting the ribosomes during protein elongation in a particular state. Certain NC sequences derived from regulatory proteins (*e.g.* SecM and TnaC) have the capacity to induce translational arrest during peptidyl transfer (Figs. 1 ▸ and 3 ▸ *b*; Wilson *et al.*, 2016 ▸). Their ability to arrest ribosomes has been exploited in structural studies of co-translational folding (Nilsson *et al.*, 2015 ▸; Cabrita *et al.*, 2009 ▸, 2016 ▸). Using cryo-EM, the stalling behaviour of NCs has been structurally characterized, revealing NC interactions with the ribosomal exit tunnel at the site of constriction (uL4/uL22), resulting in a relay back to the PTC and remodelling of the P-site to prevent further peptide-bond synthesis. Understanding the details of translational arrest also provided the first insights into the structure of the NC.

One of the first stalled NCs visualized inside the exit tunnel was the tryptophan-dependent stalling peptide derived from the TnaC protein (Seidelt *et al.*, 2009 ▸; Fig. 3 ▸ *b*). In the presence of abundant l-tryptophan levels in bacterial cells, the 24 residues of the TnaC peptide interact with the 23S rRNA nucleotides in the upper and central regions of the tunnel, and constriction-site loop residues such as Lys90 of L22 coordinate to l-tryptophan molecules to induce ribosome stalling (Seidelt *et al.*, 2009 ▸; Bischoff *et al.*, 2014 ▸; Fig. 3 ▸ *b*; 70S–TnaC). Another commonly used ribosome- stalling peptide is SecM, derived from the *E. coli* protein, which has also been analysed by cryo-EM (Bhushan *et al.*, 2011 ▸; Zhang *et al.*, 2015 ▸; Fig. 3 ▸ *b*; 70S–SecM). In bacteria, the SecM NC is translated downstream of the protein SecA, in which transient pausing by SecM is used to regulate the expression of SecA. SecA itself is a protein which forms part of the membrane-protein translocon machinery (Nakatogawa & Ito, 2001 ▸). During translation arrest, 17 amino acids corresponding to the C-terminal region of SecM interact with 23S rRNA nucleotides and the constriction-site loop residues in the exit tunnel to induce elongation arrest, as observed in the cryo-EM maps (Nakatogawa & Ito, 2001 ▸; Murakami *et al.*, 2004 ▸; Bhushan *et al.*, 2011 ▸; Zhang *et al.*, 2015 ▸). SecM stalling also gives rise to two stalling modes and two ribosome states, respectively: rotated and nonrotated states, with the likelihood of each depending on whether the P-site tRNA is covalently attached to the Gly165 or Pro166 residue at the C-terminus of SecM (Nakatogawa & Ito, 2001 ▸; Nakatogawa *et al.*, 2005 ▸; Bhushan *et al.*, 2011 ▸; Zhang *et al.*, 2015 ▸). A comparison between both TnaC and SecM NC cryo-EM maps shows both NCs forming similar interactions within the upper and central tunnel regions of the exit tunnel but differences in the NC sequences result in variations in the specific points of interaction and hence in their respective orientations within the tunnel (Fig. 4*b* ▸).

**Figure 5:**
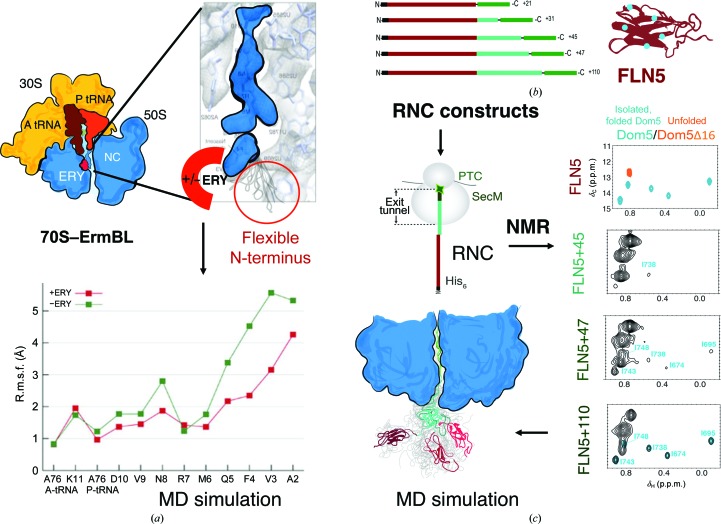
Complementary structural methods to study dynamic biological systems. (*a*) Diagram of the cryo-EM map of the ErmBL stalled NC structure bound to A-tRNA (brown), P-tRNA (dark orange) and erythromycin (ERY, red), as shown in the enlarged panel. In this panel, the ErmBL NC (blue) flexible N-­terminus is located (circled in red) near to the ERY binding pocket (red). This region was modelled in using the N-terminal peptide sequence of ErmBL with and without the ERY molecule in an all-atom MD simulation. The graph from the MD simulation (bottom panel) shows the calculated root-mean-squared fluctuations (r.m.s.f.s) in the N-terminal residues (*x* axis) with (red) and without (green) the ERY antibiotic molecule (adopted from Arenz *et al.*, 2016 ▸). (*b*) The schematic panel describes how co-translational folding of an Ig domain was studied using biochemical construct design, NMR spectroscopy and MD simulation. FLN5 RNCs (brown) were labelled at specific Ile residues (blue) with ^13^C and the RNC constructs had multiple linker lengths. The FLN6 (cyan) linkers were varied in their lengths while the FLN5 (also known as Dom5, brown) and SecM (green) peptide sequences were kept the same. This enabled tethering FLN5 (brown) on the ribosome and ‘structural snapshots’ of FLN5 emerging and folding on the ribosome to be taken by NMR spectroscopy. (*c*) Each^13^C–^1^H two-dimensional NMR spectrum shows the chemical shifts for the labelled Ile residues on FLN5 RNCs; the first spectrum in the top left panel shows an overlay of isolated and folded FLN5 (cyan peaks) and an unfolded variant (Δ16; orange peaks). This spectrum was used as a reference to map Ile residues for FLN5 RNCs at different linker lengths (two-dimensional spectra for FLN5+45, FLN5+47 and FLN5+110 below). An ensemble of FLN5 NC structures was reported using NMR spectroscopy and MD simulation (adopted from Cabrita *et al.*, 2016 ▸).

The power of cryo-EM to observe NCs has proven vital to the understanding of antibiotic-mediated ribosome stalling (Wilson *et al.*, 2016 ▸). In the case of ErmBL NCs, translational arrest in bacterial ribosomes is induced by the antibiotic erythromycin as a means of regulating the expression of the macrolide-resistant gene *ermB* (Arenz *et al.*, 2014 ▸, 2016 ▸). In the recently reported cryo-EM structure of 70S–ErmBL–RNC, the C-terminal region of the ErmBL NC was found to be well resolved, whereas tracing the remainder of the NC proved to be challenging for structural analysis, as the NC exhibited local flexibility (Figs. 3 ▸ *b* and 5 ▸ *a*; Arenz *et al.*, 2014 ▸, 2016 ▸). Previous studies suggested that erythromycin could induce translational arrest by binding to the antibiotic site and acting indirectly on the emerging ErmBL NC by redirecting its pathway along the exit tunnel (Arenz *et al.*, 2014 ▸). To probe the mechanism of action of erythromycin and its effect on the ErmBL NC, and to analyse the flexible N-terminal region of ErmBL, a combination of cryo-EM, X-ray crystallography and all-atom MD simulations was used (Arenz *et al.*, 2016 ▸). The fitted atomic structures of the NC and exit tunnel elements were taken as a starting point and residues for the N-terminal region of ErmBL were simulated in the presence and absence of erythromycin to identify the possible inter­action pathway made by ErmBL inside the tunnel that causes elongation arrest (Fig. 5 ▸ *a*). It was found that flexibility of the N-terminal ErmBL NC was decreased by erythromycin and the NC made key interactions with the tunnel wall to induce stalling. It was also suggested that the antibiotic could remodel PTC, suggesting two possible modes of interaction for NCs inside the exit tunnel (Arenz *et al.*, 2016 ▸). This study demonstrates the advantages offered by the current trend in combining near-atomic resolution cryo-EM data with molecular dynamics to describe the structure of the NC within the ribosome that would otherwise be impossible to observe by a standalone structural method. These studies also demonstrate the importance of understanding the structural basis of the interactions of the NC with the exit tunnel components and their role in co-translational protein folding.

#### 3.3.2. Visualization of NCs folding within the ribosome vestibule   

Early biochemical and biophysical studies indicated that the exit tunnel has ‘folding zones’ where emerging NCs can specifically interact with the exit tunnel elements and begin to acquire structure (Lu & Deutsch, 2005 ▸; Kosolapov & Deutsch, 2009 ▸; Woolhead *et al.*, 2004 ▸). Cryo-EM structures of RNCs are providing a direct visual assessment of how NCs are able to sample conformational space as they fold co-translationally on the ribosome. Studies by Bhushan and coworkers reported a cryo-EM structure of an 80S–RNC of dipeptidyl­aminopeptidase B which showed the NC sequence forming an α-helix at the vestibule region on the ribosome, as predicted by its strong helical propensity (Fig. 3 ▸ *c*; Bhushan *et al.*, 2010 ▸). It provided direct structural evidence that the ribosomal exit tunnel can support the formation of secondary structure.

More recent cryo-EM studies of RNCs have also revealed that the ribosome can permit the formation of simple tertiary structure at the vestibule region, as exemplified by the zinc-binding ADR1α; the NC adopts its globular domain at the centre of the vestibule, ∼80 Å away from the PTC (Fig. 3 ▸ *c*; Nilsson *et al.*, 2015 ▸). Also apparent from cryo-EM studies is that NC folding can be detected at a significant distance from the PTC within the 100 Å long tunnel. The recently reported cryo-EM structure of a spectrin domain (R16) RNC reveals a partially folded conformation of the helical bundle at the end of the vestibule, ∼95 Å away from the PTC (Fig. 3 ▸ *c*). The R16 NC forms several contacts with the vestibule region, which presumably assist in stabilizing the dynamic NC (Nilsson *et al.*, 2017 ▸; Fig. 3 ▸ *c*) and assist with its capacity to acquire structure. These emerging cryo-EM RNC structures corroborate the concept that NCs undergo folding on the ribosome.

#### 3.3.3. The majority of nascent chains fold outside the ribosome   

Despite the ability of the ribosome to support limited structure formation for the NC, the relatively limited dimensions of the ribosomal exit tunnel (20 Å at its widest), typically preclude the formation of higher-order tertiary structure: the majority of co-translational folding for large proteins occurs beyond the vestibule region of the ribosome. The highly dynamic nascent chain beyond the exit tunnel has generally eluded both cryo-EM and X-ray crystallography, whereas its study is better suited to structural methods such as NMR spectroscopy, which relies on dynamics and offers residue-specific information. Using selective isotopic labelling of the NC, RNC NMR studies have included those of the Src homology 3 (SH3) domain (Eichmann *et al.*, 2010 ▸), barnase (Rutkowska *et al.*, 2009 ▸), α-synuclein (Deckert *et al.*, 2016 ▸) and FLN5 derived from the multidomain filamin protein ABP120 (Hsu *et al.*, 2007 ▸; Cabrita *et al.*, 2009 ▸; Cassaignau *et al.*, 2016 ▸; Chan *et al.*, 2015 ▸).

More recently, merging NMR structural studies with MD simulations has become instrumental in advancing the analysis of co-translational folding: for example, to describe the first structural ensemble of an RNC (Cabrita *et al.*, 2016 ▸), which consisted of a tandem pair of immunoglobulin domains FLN5–FLN6 from the ABP120 protein (McCoy *et al.*, 1999 ▸). This ensemble showed that at a distance of 110 residues from the PTC, FLN5 was able to adopt its Ig fold. In addition, the FLN5 NC made a number of transient contacts with the 23S rRNA and several proteins within the tunnel while tethered to the ribosome through the incompletely synthesized, and thus disordered, FLN6 domain (Fig. 5*b* ▸).

Together with selective isotopic labelling to monitor dis­ordered (^15^N labelling) and structured conformations (^13^C labelling) of the NC, it was possible to describe a structural basis for co-translational folding of the FLN5 domain (Fig. 5*b* ▸; Cabrita *et al.*, 2016 ▸). Direct evidence for the folding of FLN5 was derived from two-dimensional spectra of FLN5 RNCs with selective labelling of the isoleucine side chains. Comparison of resonance chemical shifts in spectra of the FLN5 RNCs relative to an analogous folded, isolated FLN5 showed that FLN5 adopts its Ig fold when the NC is ∼45–47 residues away from the PTC of the ribosome (Fig. 5*c* ▸; FLN5+47 spectrum). Complementary biochemical studies using PEGylation indicated that the emergence of the NC occurred when FLN5 was just 34 residues away from the PTC: this separation between emergence and folding shows that the FLN5 experiences a ‘folding delay’. A per-residue analysis of two-dimensional spectra of the disordered states of FLN5 revealed residue-specific resonance broadening, which typically reflects NC dynamics and is consistent with interactions with the ribosomal surface (as predicted by simulations), demonstrating the strong influence that the ribosome has on both the structure and the dynamic properties of the NC.

## 4. Concluding remarks   

Our understanding of how proteins fold in cells is taking shape owing to remarkable developments in experimental and methodological approaches. The elucidation of the structure and function of the ribosome has come a long way through key accomplishments made by biochemical and biophysical methods, complemented by high-resolution structural tech­niques: X-ray crystallography, cryo-EM and NMR spectroscopy. The recent progress in cryo-EM and NMR has further enabled researchers to tackle structural variations in ribosomal complexes of a dynamic nature. Near-atomic structures of many functional ribosome complexes (*e.g.* RNCs) are beginning to illuminate the role of the ribosome beyond protein translation, which includes the translational arrest and co-translational folding processes.

Given the described advances in preparative biochemistry, cryo-EM, NMR and computational biology, we are now placed in a good position to answer advanced questions related to the relationship between the ribosome and the folding behaviour of an emerging NC. For instance, how does the NC folding on stalled RNC systems differ from the folding of NCs in cells in real time? Does the ribosome select and stabilize certain folding intermediates over others during co-translational protein folding? What communication occurs between chaperones and the NC during co-translational folding? Are there any specific ‘triggers’ that force the NCs to fold co-translationally before complete post-translational folding? It is clear that the highly dynamic NC undergoes significant remodelling of its structure as it folds, and addressing these complex questions requires the combination of both experimental and computational approaches to study large macromolecular assemblies (Cuniasse *et al.*, 2017 ▸).

X-ray crystallography can resolve atomic structures of molecules trapped in a rigid, crystallographic state, while NMR spectroscopy can provide atomic resolution information on both structure and dynamics on biological timescales. NMR studies of large molecules and complexes are however significantly complicated by the increased resonance linewidths associated with slower tumbling (Foster *et al.*, 2007 ▸). Cryo-EM provides a means to investigate conformational heterogeneity in molecular detail. Together, these methods present us with a magnifying glass that delivers both macroscopic and microscopic information and provides an opportunity to derive high-resolution structural and dynamic details. At different magnifications, we are able to look at different levels of structural detail of molecular complexes that enable us to understand biological function. This provides a powerful hybrid structural biology framework to study large macromolecular complexes such as RNCs and advance our understanding of the fundamental question of protein folding.
